# Retracted: Protective Effect of D-Limonene against Oxidative Stress-Induced Cell Damage in Human Lens Epithelial Cells via the p38 Pathway

**DOI:** 10.1155/2022/9862315

**Published:** 2022-05-29

**Authors:** Oxidative Medicine and Cellular Longevity

*Oxidative Medicine and Cellular Longevity* has retracted the article titled “Protective Effect of D-Limonene against Oxidative Stress-Induced Cell Damage in Human Lens Epithelial Cells via the p38 Pathway” [1]. As raised on PubPeer [2], the article was found to contain errors with in Figures 1(a, d, e), and Figure 2(a). The details are as follows:

- In Figure 1, there were errors in the numbering of the *x*-axes, with 0 missing and inappropriate scaling
- In Figure 2(a), the flow cytometry dot plot representing the control has regions of unexpected similarity with the dot plot for H_2_O_2_ + D-limonene 2000 *μ*M

We asked the authors for their explanation and to provide the uncropped and unadjusted images underlying all the figure. The authors explained that they believed that the result for 0 *μ*M was not meaningful, so they did not show this value in Figure 1. The authors said they provided the incorrect picture in the control group of Figure 2(a) due to an oversight and provided a corrected figure with the control group. The authors' response is in the supplementary materials, including corrected figures. However, this did not satisfy our concerns and the Editorial Board recommended retraction.

The authors agree with this retraction.

## Supplementary Materials

Supplementary Materials Authors' response to the editor, included data for Figure 1 and a revised Figure 2.
