# Supplementary material for: Retracted: Protective Effect of D-Limonene against Oxidative Stress-Induced Cell Damage in Human Lens Epithelial Cells via the p38 Pathway
Source: Oxid Med Cell Longev. 2022 May 29;2022:9862315. doi: 10.1155/2022/9862315 (PMC9168100; doi:10.1155/2022/9862315)
Supplement: Supplementary Materials — Authors' response to the editor, included data for Figure 1 and a revised Figure 2. [file 9862315.f1.docx]

**Figure 1 has errors in the numbering of the X-axis and/or Y-axis.**

We thank the reader for the advice, in this experiment, we test 0, 25, 50, 100, 200, 400, 800 𝜇MH_2_O_2_ for 12, 24, and 48 h by an MTT assay, didn’t test the concentration of 10 𝜇M.

The original data were shown in the below tables. In this article, our purpose is to explore the effect of different concentrations of drug (Figure1 a--H_2_O_2_, Figure 1d—D-limonene, Figure 1e—H_2_O_2_ and D-limonene) on cell viability. Although we measured cell viability without drug intervention during the experiment, we thought that the result of 0 was not meaningful for the experiment, so we did not show the value in the results.

Figure 1a in article	Supplementary picture

Figure 1d in article	Supplementary picture

Figure 1e in article	Supplementary picture

**In Figure 2a, the flow cytometry dot plot representing the control seems similar to another dot plot (H_2_O_2_ + D-limonene 2000 µM). The two blue circles mark the areas that are most different.**

Thanks the reader very much for pointing out it and am very sorry for putting the wrong picture in the control group because of my careless, I double checked and find out the original picture and replaced the correct picture with the control group.

Figure 2a in article

Control	100 𝜇MH_2_O_2_	H_2_O_2_+D-limonene 500 𝜇M

H_2_O_2_+D-limonene 1000𝜇M	H_2_O_2_+D-limonene 2000𝜇M

the original, unedited, images for Figure 2a

Revised Figure 2a
